# PIGA Mutations and Glycosylphosphatidylinositol Anchor Dysregulation in Polyposis-Associated Duodenal Tumorigenesis

**DOI:** 10.1158/1541-7786.MCR-23-0810

**Published:** 2024-03-28

**Authors:** Elena Meuser, Kyle Chang, Angharad Walters, Joanna J. Hurley, Hannah D. West, Iain Perry, Matthew Mort, Laura Reyes-Uribe, Rebekah Truscott, Nicholas Jones, Rachel Lawrence, Gareth Jenkins, Peter Giles, Sunil Dolwani, Bilal Al-Sarireh, Neil Hawkes, Emma Short, Geraint T. Williams, Melissa W. Taggart, Kim Luetchford, Patrick M. Lynch, Diantha Terlouw, Maartje Nielsen, Sarah-Jane Walton, Andrew Latchford, Susan K. Clark, Julian R. Sampson, Eduardo Vilar, Laura E. Thomas

**Affiliations:** 1 Division of Cancer and Genetics, School of Medicine, Cardiff University, United Kingdom.; 2 Department of Clinical Cancer Prevention, The University of Texas MD Anderson Cancer Center, Houston, Texas.; 3 Department of Gastroenterology, Cwm Taf Morgannwg University Health Board, Llantrisant, United Kingdom.; 4 Institute of Life Science 1, Swansea University, Swansea, United Kingdom.; 5 Division of Population Medicine, School of Medicine, Cardiff University, United Kingdom.; 6 Department of Gastroenterology, Swansea Bay University Health Board, Swansea, United Kingdom.; 7 Department of Cellular Pathology, Swansea Bay University Health Board, Swansea, United Kingdom.; 8 Department of Pathology and Laboratory Medicine, The University of Texas MD Anderson Cancer Center, Houston, Texas.; 9 Cellesce Limited, Cardiff Medicentre, Cardiff, United Kingdom.; 10 Department of Gastroenterology, Hepatology and Nutrition, UT MD Anderson Cancer Center, Houston, Texas.; 11 Clinical Cancer Genetics Program, UT MD Anderson Cancer Center, Houston, Texas.; 12 Leiden University Medical Center (LUMC), Department of Clinical Genetics, Leiden, The Netherlands.; 13 St Mark's Centre for Familial Intestinal Cancer, St Marks Hospital, London, United Kingdom.; 14 Department of Surgery and Cancer, Faculty of Medicine, Imperial College, London, United Kingdom.

## Abstract

The pathogenesis of duodenal tumors in the inherited tumor syndromes familial adenomatous polyposis (FAP) and MUTYH-associated polyposis (MAP) is poorly understood. This study aimed to identify genes that are significantly mutated in these tumors and to explore the effects of these mutations. Whole exome and whole transcriptome sequencing identified recurrent somatic coding variants of phosphatidylinositol N-acetylglucosaminyltransferase subunit A (*PIGA*) in 19/70 (27%) FAP and MAP duodenal adenomas, and further confirmed the established driver roles for *APC* and *KRAS*. PIGA catalyzes the first step in glycosylphosphatidylinositol (GPI) anchor biosynthesis. Flow cytometry of *PIGA*-mutant adenoma-derived and CRISPR-edited duodenal organoids confirmed loss of GPI anchors in duodenal epithelial cells and transcriptional profiling of duodenal adenomas revealed transcriptional signatures associated with loss of *PIGA*.

**Implications:**

*PIGA* somatic mutation in duodenal tumors from patients with FAP and MAP and loss of membrane GPI-anchors may present new opportunities for understanding and intervention in duodenal tumorigenesis.

## Visual Overview

**Figure:**
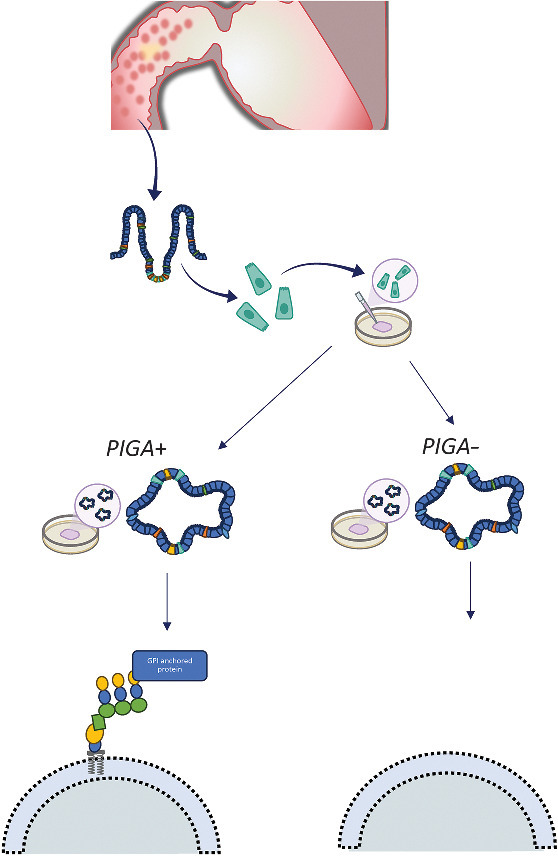

## Introduction

The inherited gastrointestinal tumor syndromes familial adenomatous polyposis (FAP, OMIM #175100) and MUTYH-associated polyposis (MAP, OMIM #608456) are associated with increased risks of colorectal cancer and extra-colonic manifestations including adenomas and carcinomas of the duodenum (1). Successful management of colorectal disease has improved the life expectancy of patients, but morbidity and mortality associated with duodenal disease, which typically occurs later, remain significant (2). In FAP, the cumulative risks of duodenal adenomas and cancer at 75 years of age have been estimated at 88% and 18%, respectively (2). A recent cross-sectional study identified duodenal adenomas in 14.5% of MAP patients at their first endoscopy (median age 51 years) with a further 21.1% of previously unaffected individuals developing adenomas during a median follow-up of 4 years (1). Endoscopic surveillance of the duodenum is recommended in those with FAP or MAP with the aim of preventing duodenal cancer (3). Spigelman staging was developed to determine endoscopy intervals and timing of surgical intervention for duodenal disease in FAP (4). Its use is currently recommended in guidelines for clinical management of both FAP and MAP, although its suitability for determining endoscopic surveillance intervals in MAP requires further clarification. Duodenal polyposis in patients with FAP appears to progress slowly through the Spigelman stages 0 to IV, with a higher risk of cancer development in patients with adenomas of large size, villous appearance, and high-grade dysplasia (2). Around half of FAP patients who develop duodenal cancer do not have stage IV disease at the last endoscopy prior to diagnosis of cancer (5). In MAP, the natural history of duodenal polyposis is poorly defined, but cancer can occur even in the context of minimal polyposis (1). Endoscopic management is the mainstay of treatment, with duodenectomy indicated for those who are not manageable endoscopically. Surgical intervention carries significant morbidity and mortality risks. There is a clinical need for pharmacologic prevention or treatment of duodenal adenomas that has not yet been achieved.

A better understanding of the molecular basis of duodenal tumorigenesis in FAP and MAP is needed to discover targetable pathways for effective cancer interception strategies. Significant differences exist between the patterns of somatic mutation in duodenal and colorectal adenomas in patients with FAP and MAP, including recurrent mutation of *WTX* (*AMER1*) in colorectal but not duodenal adenomas (6). This study aimed to identify genes harboring recurrent somatic coding mutations in duodenal adenomas in patients with FAP or MAP, to provide insights into the molecular mechanisms of duodenal tumorigenesis.

## Materials and Methods

### Patients and tissue samples

All participants had MAP or FAP confirmed by genetic testing and were recruited from the All Wales Medical Genomics Service, Cardiff or St Mark's Hospital, London, to the study center at Cardiff University (CU) or at the High-Risk Familial Gastrointestinal Clinic at The University of Texas MD Anderson Cancer Center (MDACC). The study was approved by UK NHS Research Ethics Committees (reference 10/MRE093 and 15/WA/0075) and the Institutional Review Board of MDACC (protocol number PA12–0327). All participants provided written informed consent and the study was completed in accordance with the Declaration of Helsinki. Polyp and normal mucosa biopsies were obtained at routine surveillance upper gastrointestinal endoscopy or duodenal surgery, and either snap frozen with liquid nitrogen and stored at −80°C, collected in RNALater solution (Qiagen) and stored at 4°C, or collected in Hibernate A medium (Thermo Fisher Scientific) and processed for organoid establishment (Supplementary Materials and Methods). A 4 mL blood sample was also collected to facilitate discrimination of constitutional from somatic genetic variants. Modified Spigelman stage of duodenal polyposis was calculated as described by Saurin and colleagues (3). Histopathologic classification, including the degree of dysplasia and proportions of neoplastic cells, were determined from haematoxylin and eosin-stained sections by specialist GI pathologists (E.S., G.T.W. and M.W.T.). Microdissection of adenomas was not undertaken with potential impact on the detection of variants present only in neoplastic cells. Full clinicopathologic information is in Supplementary Table S1. Cores from an FFPE block of a MAP duodenal carcinoma and matched colorectal adenoma from the same patient were obtained from PALGA, the Dutch nationwide pathology databank.

### Nucleic acid extraction and QC

DNA extraction from whole blood samples was carried out using an automated facility (Hamilton) or using standard protocols (7). Extraction of DNA and RNA from tissue biopsies utilized the AllPrep DNA/RNA/Protein Mini Kit (Qiagen) as per the manufacturer's recommendations. DNA from FFPE cores from a MAP duodenal carcinoma and matched colorectal adenoma, were extracted with the Covaris truXTRAC FFPE DNA microTUBE Kit. All extracted RNA underwent quality assessment on a Bioanalyzer 2100 platform (Agilent) with an RNA 6000 Nano Kit (Agilent). Samples with RIN values >7 (>4 for MDACC) were used (8). Extracted DNA samples were quantified using a Qubit fluorometer (Thermo Fisher Scientific).

### Next-generation sequencing

Whole-exome sequencing (WES) of duodenal adenomas, MAP duodenal carcinoma and matched constitutional DNA from peripheral blood (or matched colorectal adenoma for the duodenal carcinoma), was undertaken using the SureSelect Human 50 Mb Capture Kit (Agilent) or the Nextera Rapid Capture Exomes Kit (Illumina) on Illumina HiSeq2500 platforms using a 75 bp paired-end dual index read format. Libraries were prepared from approximately 300 ng of starting DNA. Mean WES coverage was 130×, with >70% of the target covered at least 50×.

Whole transcriptome sequencing (WTS) was achieved by RNA sequencing (RNA-seq) of adenomas and normal mucosa from the CU series. Samples were sequenced as technical triplicates by the Wales Gene Park Genomic facility (Cardiff University) from approximately 120 ng RNA starting material, using the TruSeq Stranded Total RNA Kit with Ribo-Zero Gold (Illumina) on an Illumina HiSeq 2500 platform in a 75 bp paired-end dual index read format. A mean of 36 million (M) read pairs per sample were generated, of which on average 76.7% (27 M pairs) aligned uniquely to the genome. RNA-seq of the MDACC FAP duodenal samples was performed on an Illumina HiSeq 2000 sequencer at The University of Texas MD Anderson Sequencing Core Facility as described previously (9). Samples were sequenced to a mean of 55 M read pairs per sample, of which on average 86% (47 M pairs) aligned uniquely to the genome. For both data sets, over 80% of reads mapped to known exons (Supplementary Fig. S1).

### Whole exome data processing

All sequencing reads were processed as per the GATK best practice workflow for somatic variant calling. In brief, sequencing reads underwent quality assessment using FastQC and were mapped against human reference hg19 using BWA-MEM. Sorted indexed .bam files were created with samtools. Mappings from multiple runs were merged with Picard MergeSamFiles and read duplicates were flagged with Picard MarkDuplicates. Realignment around indels, recalibration of base quality values, and assessment of cross-sample contamination was achieved with GATK. Somatic variant and indel calling for every adenoma/normal pair were undertaken using the MuTect2 algorithm, a combination of the original MuTect and GATK's HaplotypeCaller, which is particularly powerful for variant calling in heterogeneous tissue samples. MuTect2 was run on default parameters.

### Validation of somatic variants

Interrogation of the performance and fidelity of the BWA-MuTect2 pipeline was undertaken in a two-tiered approach based on Sanger sequencing followed by droplet digital PCR (ddPCR; Supplementary Materials and Methods). The Sanger sequencing limit of detection was determined to be 5.7% variant allele frequency (VAF), above this, all mutations in the validation subset which were called by the BWA-MuTect2 pipeline were confirmed. For mutations below the Sanger detection limit, ddPCR was employed to examine a subset of variants called at defined VAFs. Three mutations called at 1% VAF and three further mutations at 0.6% VAF were analyzed, all of which were confirmed. On the basis of these results, no VAF-based filtering was applied.

For each of the somatic coding *APC, KRAS*, and phosphatidylinositol N-acetylglucosaminyltransferase subunit A (*PIGA*) variants identified by WES, validation of the variant, and confirmation of absence from the constitutional DNA or matched normal mucosa (where available), was undertaken by direct Sanger sequencing as described previously (6). Confirmation of the somatic variants in matched WTS data was possible for 21 samples which had been subjected to both WES and WTS (FAP *n* = 9, MAP *n* = 12; Supplementary Table S1). Variants were identified from WTS data through targeted manual inspection of .bam files in Integrative Genomics Viewer (IGV, Broad Institute). This was followed by validation of the variant as described above, and confirmation of absence from constitutional DNA and matched normal mucosa (where available) by direct sequencing of DNA/cDNA or analysis of matched WES data.

### Somatic driver gene analysis

Exome-wide somatic variants were analyzed using R software. To identify potential tumor driver genes that were subject to positive selection we first used the “MutSigCV” algorithm (10), accessed via GenePattern using MAF files generated using Oncotator with a FDR cut off of *q* ≤ 0.01. MutSigCV results were compared with TCGA-based pan cancer drivers using the R package “maftools.” Second, dNdScv (11) was run using; max_coding_muts_per_sample = 5000 and max_muts_per_gene_per_sample = 20 and genes with a *q* of <0.05 were classified as being under positive selection.

### Transcriptome data processing and differentially expressed gene discovery

Sequencing files underwent quality assessment using FastQC and were aligned to the human genome (hg19) with STAR in a two-stage mapping process. Quality assessment of the resultant .bam files was carried out using Samtools. Reads were counted with HTSeq-count in union mode, retaining only those reads that mapped uniquely. SARTools using the DESeq2 package was used to generate PCA analysis and calculate differentially expressed genes (DEG) using default parameters. Adjusted *P* values (*P*_adj_) were calculated using Benjamini–Hochberg false discovery correction. DEGs were filtered using an *P*_adj_ ≤ 0.05 and a LFC ≥±1.5. Pathway analyses of DEGs were carried out using gProfiler with a g:SCS threshold *P*_adj_ ≤ 0.05, term-size limited to 1,000. GO terms were reduced for visualization with REVIGO. Validation was carried out using qRT-PCR and IHC staining in a subset of tissue samples that had undergone RNA-seq (Supplementary Materials and Methods).

### Sequencing of *PIGA* in colorectal adenomas

Sanger sequencing of all coding exons of *PIGA* was completed in DNA from 29 colorectal adenomas (FAP *n* = 15, MAP *n* = 14) from 7 patients (FAP *n* = 4, MAP *n* = 3). Sequencing was completed using MegaMix-Gold (Microzone) polymerase mix, ExoSAP enzymatic reaction clean-up (NEB and Affymetrix, respectively), BigDye terminator ready mix v1.1 (Thermo Fisher Scientific) and HiDi Formamide (Thermo Fisher Scientific). PCR was completed on an AlphaCycler4 (PCRmax) prior to sequencing on ABI3730 DNA sequencers (ABI). Sequencing data analysis was carried out using “Sequencher” software (GeneCodes).

### Patient derived organoids

Eleven organoid lines were used in this study. Nine patient-derived organoid lines were established from duodenal biopsies from 7 patients with FAP (Supplementary Table S2; Supplementary Materials and Methods). These included two lines from macroscopically and histologically normal mucosa, and seven from histologically confirmed duodenal adenomas including two (from a male patient) with somatic *PIGA* mutations (c.915dupT; p.L306fs*7 and c.945C>G; p.C314W) and five that were wild-type for *PIGA*. In addition, *PIGA* was targeted in one of the *PIGA* wild-type adenoma lines by CRISPR (Synthego) to produce two additional organoid lines with a complete somatic *PIGA* knockout. Confirmation of knockout and estimation of knockout efficiency was completed by direct sequencing and analysis using ICE (Synthego).

### Assessment of GPI-anchor abundance using flow cytometry

The abundance of GPI anchors was assessed by flow cytometry in the 11 duodenal organoid lines. FLAER is an Alexa Fluor 488-labeled variant of bacterial aerolysin toxin, which binds specifically to mammalian GPI anchors (VHbio), discriminating between cells with and without these anchors. Flow cytometry using FLAER was completed using a BD FACS Canto II (Central Biotechnology Services, CU) and BD FlowJo 10 software (Supplementary Materials and Methods). Each organoid line was analyzed in technical triplicates. Lines 1 to 4, 8, and 9 were also further repeated three times.

### Statistical analyses

Student *t* test was used to compare two independent means from normally distributed data, whereas Mann–Whitney *U* test was used for nonnormally distributed data. Fisher exact test was used to analyses 2 ×2 contingency tables. For large and imbalanced comparisons chi^2^ with Yates correction was used. All analyses were undertaken using R, considering a *P* value of ≤0.05 as statistically significant.

### Nomenclature

All genomic coordinates refer to human genome assembly GRCh37 (hg19). Where specifically named, mutational nomenclature refers to the following reference sequences: *APC* (RefSeq NM_000038.6), *KRAS* (NM_004985.5) and *PIGA* (NM_002641.4).

### Data availability

Genomic datasets are available through EGA under accession: EGAD00001009332 and in GEO under accession ID GSE189035. Other data are available from the corresponding author upon request.

## Results

### Patients and samples

Duodenal adenoma and normal mucosal biopsies were obtained from 41 patients, 25 with FAP and 16 with MAP (Supplementary Tables S1 and S3). Patients with MAP were older than those with FAP (*P* = 0.0004) and had less severe duodenal disease, evidenced by lower Spigelman stage (*P* = 0.002; Supplementary Table S3). The biopsies included 70 adenomas (44 FAP, 26 MAP) from 29 different patients (21 FAP, 8 MAP) and 24 normal mucosa samples (16 FAP, 8 MAP; Supplementary Table S1).

### Genomic analysis of FAP and MAP duodenal adenomas by exome sequencing

Analysis of 48 duodenal adenoma exomes by both MutSigCV and dNdScv showed that somatic mutations in *APC* and *PIGA* were significantly overrepresented (*q* < 0.01) in FAP duodenal adenomas, and *APC*, *PIGA*, and *KRAS* mutations in MAP duodenal adenomas (*q* < 0.01; Supplementary Table S1). Although *APC* and *KRAS* are well established driver genes in gastrointestinal tumorigenesis, it was notable that *PIGA* mutations were identified in 8/24 FAP adenomas and 8/24 MAP adenomas and that 14 of the 16 mutations were predicted to be truncating. Where multiple adenomas were analyzed from the same patient, whole-exome mutational profiles were distinct.

Next, we increased the number of adenoma samples available for somatic mutation analysis by interrogation of WTS data from 22 additional duodenal adenomas for which WES data were unavailable (20 FAP and 2 MAP), identifying three further somatic *PIGA* mutations, one truncating and two missense, all in FAP adenomas, and further *APC* and *KRAS* mutations (Supplementary Table S1). For 21 adenomas with both WES and WTS data, the latter was used to confirm the *APC*, *PIGA*, and *KRAS* mutations identified by WES, with concordant findings in all cases.

Variants identified through WES analysis, arising in other genes outside of *APC, KRAS*, and *PIGA*, which may be putatively associated with intestinal tumorigenesis, can be seen in Supplementary Tables S4 and S5. The genes in which these variants occur, were not significantly recurrently mutated using the analyses described here.

**Figure 1.**
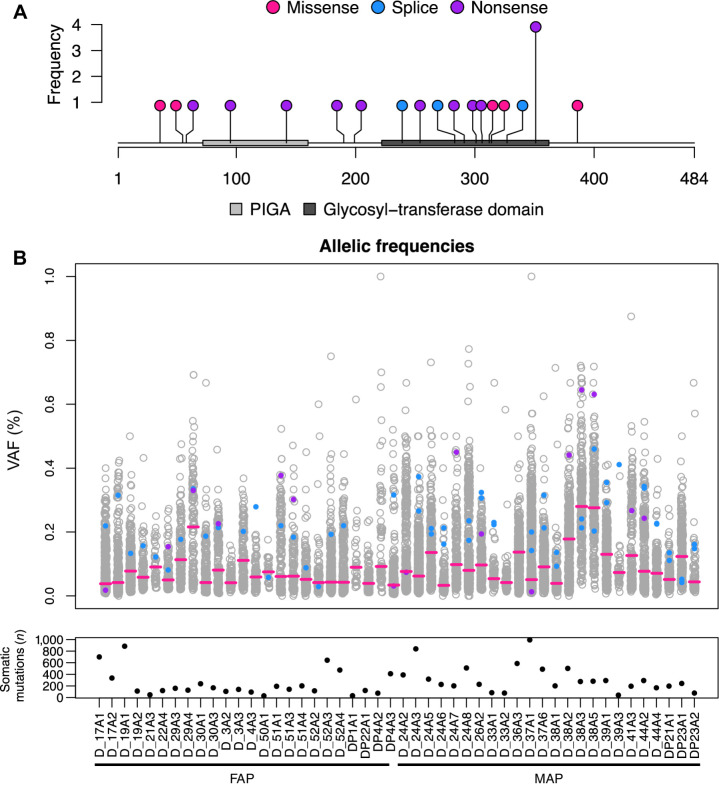
**A,** *PIGA* mutations identified in 19 duodenal adenomas and two duodenal adenoma patient-derived organoid lines. Relative location in comparison with known protein domains is shown. **B,** The VAF of four types of somatic mutation (exonic − synonymous/missense + indels/nonsense; all other mutations) for all adenomas analyzed from FAP and MAP patients. For each duodenal adenoma sample, all somatic mutations are shown as circles indicating the allelic frequency of the respective mutant alleles. The median VAF per sample is represented with a red bar. The APC VAFs are indicated in blue and PIGA in purple. The total number of somatic mutations in each adenoma sample is displayed in the lower graph.

In total, across the 70 duodenal adenoma samples from FAP and MAP patients, somatic coding *PIGA* mutations were identified in 19 (27%), including those from both male (*n* = 6) and female (*n* = 8) patients (Fig. 1). Fifteen of 19 (79%) were truncating and four (22%) were missense variants, all with CADD scores >20, suggesting pathogenicity (Supplementary Table S1). No somatic synonymous variants were detected. Eleven of 19 (58%) were in the glycosyltransferase domain, with S351X found in 4 of 19 (21%) of the adenomas. No variants were found in the transmembrane domain (Fig. 1A).

Although most (12/19) adenomas with *PIGA* mutations were tubular adenomas (TA) with low-grade dysplasia (LGD), 3/5 adenomas with high-grade dysplasia in the study had *PIGA* mutations (*P* = 0.119) as did 4/9 with tubulovillous or villous histology. *KRAS* mutations, in comparison, were found in 20/70 (29%) adenomas, all but one of which were TAs with LGD. Four adenomas had both *PIGA* and *KRAS* mutations and were all TAs with LGD (Supplementary Table S1).

### Timing of *PIGA* mutation and mutant allele frequencies in tumorigenesis

Consistent with the mutator phenotype associated with MUTYH-deficiency (6, 12), adenomas from MAP patients had a higher burden of somatic mutations than those from FAP patients (Supplementary Fig. S2), although the differences did not reach statistical significance when all types of mutation were combined. The total number of somatic mutations per Mb (Muts/Mb) was 3.6 Muts/Mb in FAP duodenal adenomas with *PIGA* mutations, compared with 3.1Muts/Mb, (*P* = 0.57) in FAP duodenal adenomas without *PIGA* mutations. In MAP adenomas, the Muts/Mb was also not significantly different between *PIGA* mutated and nonmutated adenomas (4.7Muts/Mb vs. 4.2Muts/Mb, *P* = 0.83). The median mutant allele frequency of somatic variants detected in duodenal adenomas was 8.4% (range: 3.3%–28%), indicative of an admixture of neoplastic and nonneoplastic cells in the biopsies (Fig. 1B; Supplementary Table S1). Although the *PIGA* mutant allele frequency varied considerably between adenomas, it was above the median mutant allele frequency for the corresponding adenoma in most cases, a pattern similar to *APC*, and consistent with occurrence early in tumorigenesis (Fig. 1B; Supplementary Table S1).

### Somatic mutation of *PIGA* in FAP and MAP colorectal adenomas and duodenal carcinoma

Sequencing of the *PIGA* open reading frame in 29 colorectal adenomas (FAP *n* = 15, MAP *n* = 14) from 7 patients (FAP *n* = 4, MAP *n* = 3) revealed no truncating variants and only one (3.4%) missense variant (c.128A>T, p.Tyr43Phe, CADD score 22.3, “Tolerated” with SIFT and “Benign” with Polyphen). This variant was not observed in any of the duodenal adenomas analyzed. Somatic *PIGA* mutations occurred significantly less frequently in colorectal than duodenal adenomas in patients with FAP or MAP (*P* < 0.001).

A somatic coding *PIGA* mutation was identified in the single MAP duodenal carcinoma analyzed (c.282delGA; p.K95SfsX32) and was absent from a colorectal adenoma from this patient, indicating it was a somatic event. A different variant at the same codon was identified in an adenoma from another MAP patient (Supplementary Table S1).

### Assessment of GPI-anchor abundance using flow cytometry

**Figure 2.**
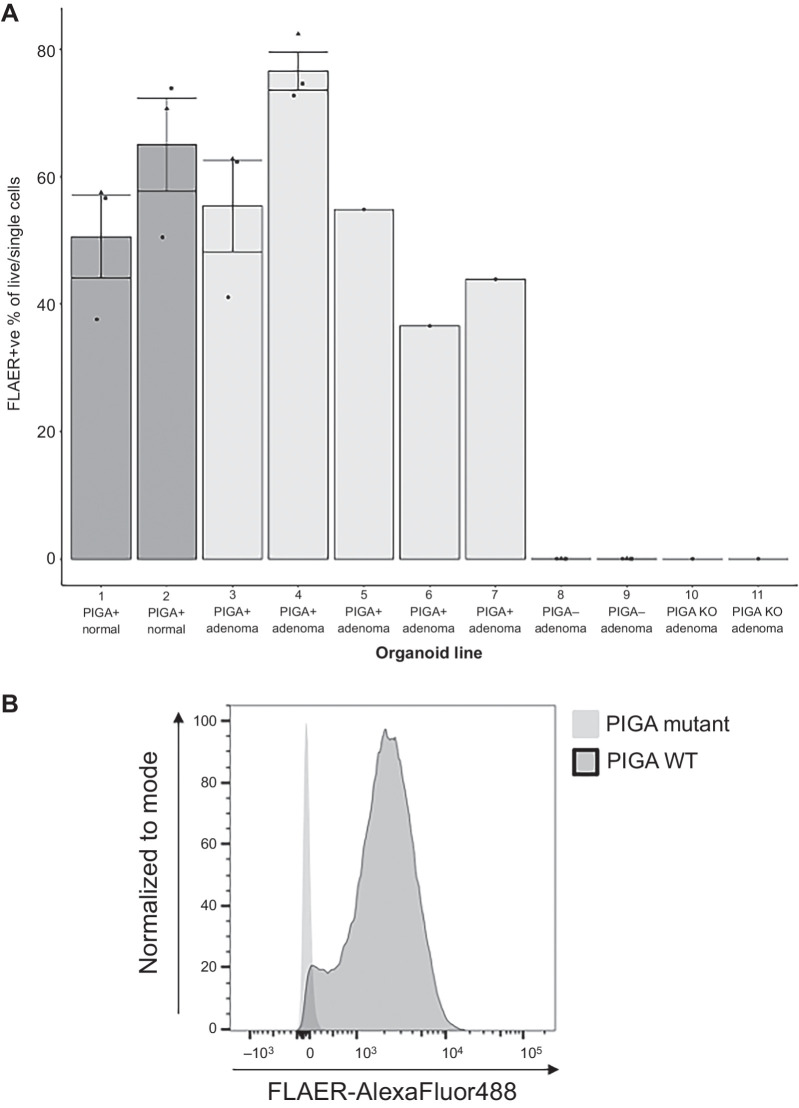
Flow cytometry analysis of FLAER positive cells in 11 FAP duodenal organoids with and without somatic *PIGA* variants. Fluorescence from FLAER is detected using the FITC-A channel (emission:475–650 nm). **A,** Abundance of FLAER positive cells detected in organoid lines with and without *PIGA* somatic mutations demonstrating lack of FLAER positive cells in *PIGA* mutant lines, indicating loss of GPI-anchors (Supplementary Table S2). **B,** Representative histogram of FITC-A/FLAER (selecting for FLAER positive cells), light grey = wild-type adenoma, dark grey = *PIGA* mutant adenoma. Repeats are denoted with a black triangle, circle, and square.

PIGA catalyzes the first step of the GPI biosynthetic pathway. We analyzed 11 duodenal organoid lines by flow cytometry using a bacterial fluorescein-labeled proaerolysin (FLAER) that binds to GPI-anchors (Fig. 2; Supplementary Table S2; Supplementary Fig. S3). Organoid lines which were wild type for *PIGA* (*n* = 7, Fig. 2A lines 1–7) had a mean percentage of FLAER positive single/live cells of 60.7%, SD = 13.8. In contrast, virtually no FLAER positive cells were present in *PIGA* mutant organoid lines (*n* = 4, Fig. 2A lines 8 and 11, 0.0239%, SD = 0.0115, *P* < 0.001), indicating an absence of GPI anchors. Two of the mutant lines contained naturally occurring somatic *PIGA* point mutations and two (lines 10 and 11) were generated by CRISPR from one of the *PIGA* wild-type adenoma-derived organoids (line 7; Fig. 2; Supplementary Table S2). In Synthego ICE analysis, the indel percentage and knockout scores were 100 for both edited lines. The loss of FLAER positive cells following CRISPR supports the loss of GPI anchors being causally linked to *PIGA* mutation. The proportion of FLAER positive single/live cells in *PIGA* mutant lines was the same as that of the unstained controls (∼0%; Fig. 2B).

### Integration of genomic and transcriptomic data

*PIGA* has not previously been significantly associated with any cancer. Differential transcriptional activity, indicating altered cellular processes, was assessed in matched WES and WTS data from 20 duodenal adenomas (*n* = 10 FAP and *n* = 10 MAP) stratified into those with *PIGA* somatic coding mutations (*n* = 7, 3 FAP, 4 MAP) and those without (*n* = 13, 6 FAP, 7 MAP). Normalization, DEG discovery, and validation were completed as described in the Supplementary Materials and Methods (Supplementary Fig. S4). Genes with a *P*_adj_ < 0.01 and a LFC ≥ 1.5 were considered DEGs.

**Figure 3.**
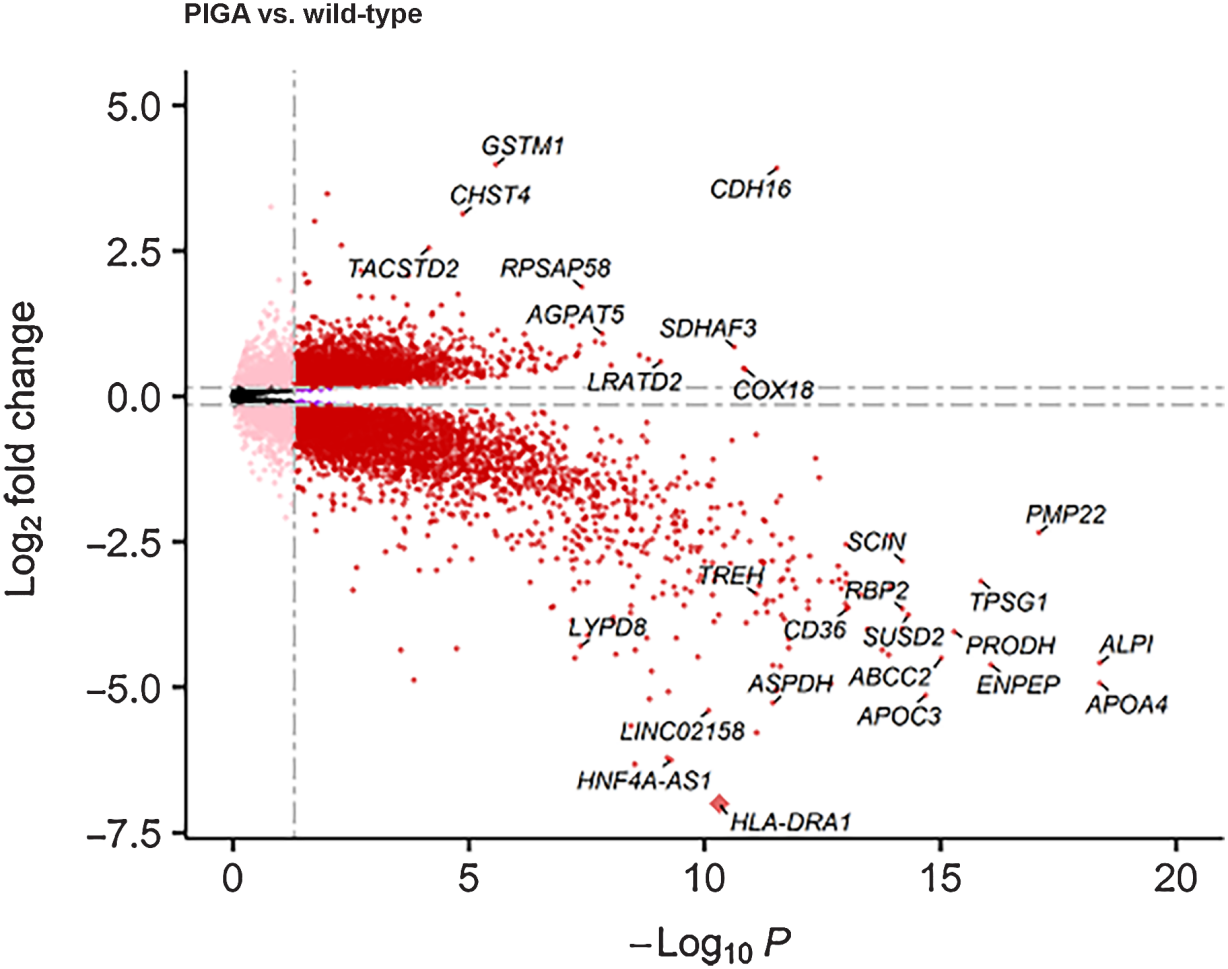
Volcano plot of DEGs of FAP and MAP patients with and without PIGA mutation. Genes have been filtered to remove those with a mean expression ≤10 read counts. Red dots indicate DEGs with *P*_adj_ ≤ 0.01 at ≥±1.5 LFC. Purple dots indicate genes with a *P*_adj_ ≤ 0.01 but ≤±1.5 LFC. Nonsignificant genes are greyed out. Highly DEGs are highlighted, including *CD36*, *TREH*, and *LPD8*. *HLA-DRA1* has been transformed (diamond) from −10.3 LFC to fit on the plot.

This identified 1,413 DEGs (with HGNC gene IDs; Fig. 3), with 88 genes upregulated and 1,325 genes downregulated. Sixteen of these DEGs (all downregulated in *PIGA* mutant adenomas) were known GPI-APs. These included *CD36* (−3.66), Trehalase (*TREH*: −3.40), LY6/PLAUR Domain Containing 8 (*LYPD8*: −4.29), and intestinal alkaline phosphatase (*ALPI*: −4.58). Gene ontology analysis of cellular components highlighted an enrichment of gene ontologies associated with the brush border and apical plasma membrane (both *P* < 0.001). An enrichment of DEGS associated with biological processes including intestinal absorption, salt transmembrane transporter activity, organic anion transport, and lipid transport were also identified (all *P* < 0.001; Supplementary Fig. S5).

## Discussion

Previous genomic analyses have highlighted differences in the pattern of molecular alterations in duodenal and colorectal adenomas. Notably, there is a distinct *APC* mutation cluster region in duodenal adenomas compared with that observed in colorectal tumours and *WTX* mutations that were observed recurrently in exome sequencing of colorectal adenomas (12), were absent from duodenal adenomas (6). These observations suggest that there are subtle but significant differences in WNT-related signaling in colorectal and duodenal tumorigenesis. Here, we identify a novel and apparently specific role for *PIGA* in duodenal tumorigenesis, associated with loss of GPI anchors in duodenal epithelial cells.

*PIGA* encodes a 484 amino acid catalytic subunit of a protein complex, which is required for the synthesis of N-acetylglucosaminyl phosphatidylinositol (GlcNAc-PI), the first step in glycophosphatidylinositol (GPI) anchor synthesis (13). In mammals, there are over 150 GPI-anchored proteins (GPI-AP), that may attach to the luminal/extracellular leaflet of the membrane bilayer, conferring specific functions and trafficking of the proteins. At least 31 genes are known to be involved in GPI biosynthesis, and pathogenic variants in at least 22 of those genes have been linked to human disease (14). Constitutional mutation of *PIGA* is associated with the rare disorder; PIGA-related congenital disorder of glycosylation (PIGA-CDG), resulting in high mortality in childhood and a largely neurologic phenotype (13). Somatic mutations affecting *PIGA* in hematopoietic stem cells lead to the rare auto-immune disease paroxysmal nocturnal hemoglobinuria (PNH; ref. 15). As *PIGA* is located on the X chromosome, somatic mutation of a single allele is sufficient to impair function in males, and in females where one X chromosome is inactivated by lyonization. *PIGA* function cannot apparently be compensated for by any other member of the enzyme complex and thus loss of *PIGA* manifests in a deregulation of GPI-anchored proteins. GPI-anchored proteins and components of the GPI anchor biosynthesis pathway including PIGA are prognostic biomarkers in cancer (16). The ease of identifying *PIGA* mutant cells by flow cytometry (15) has led to the development of routine flow-based mutation detection assays for PNH and for mutagenicity in drug safety assessment (16).

The availability of genomic data to provide insight into the molecular evolution of duodenal adenomas into cancer is absent in FAP and MAP, and rare in sporadic tumors. *PIGA* was not identified as a significantly and recurrently mutated gene in 12 sporadic duodenal adenocarcinomas analyzed by Yuan and colleagues (17), although a different study of 21 sporadic duodenal carcinomas revealed one *PIGA* variant (p.Q58X; ref. 18), indicating that mutation of *PIGA* may also be involved in duodenal carcinoma development outside of the context of FAP and MAP. Previous studies on FAP- or MAP-associated colorectal adenomas identified no *PIGA* variants (12) and a targeted screen of *PIGA* in colorectal adenomas from FAP and MAP patients in the current study identified only a single *PIGA* variant that was not likely to cause loss of function.

*PIGA* mutations were also rare among the 10,967 cancers in the TCGA pan cancer dataset (65 variants, 0.6%) and in PCAWG (106 exonic variants/19,729 donors, 0.54%). Notably, the TCGA pan-cancer dataset includes one sporadic ampullary cancer, which harbors a truncating *PIGA* mutation (C251Sfs*31). In contrast to our findings in duodenal adenomas where most *PIGA* mutations were truncating, in the TCGA and PCAWG datasets, 92% and 83% of variants respectively, are missense variants. Constitutional variants affecting *PIGA* in the PIGA-CDG syndrome show a similar distribution to the somatic variants we observed in duodenal adenomas (14). The absence of synonymous somatic *PIGA* variants in the adenomas studied here, suggests positive selection for nonsynonymous variants (11).

The VAFs associated with *PIGA* variants in this study suggest that the variants may arise early in duodenal adenoma development in a proportion of already initiated adenomas, perhaps following *APC* initiating events but preceding those affecting other known tumor drivers such as *KRAS*. The presence of *PIGA* variants in 7/19 (37%) higher grade lesions (TA with high-grade dysplasia and tubulovillous adenomas) and a duodenal carcinoma from a MAP patient, might also suggest a role for *PIGA* in tumor progression. Study of a more extensive sample series, including advanced adenomas and cancers could investigate this possibility further.

Flow cytometry analysis of two FAP adenoma-derived duodenal organoid lines indicated that their *PIGA* mutations resulted in complete loss of GPI anchors on the cell membrane. In contrast, some constitutional *PIGA* mutations that cause *PIGA*-CDG in liveborn children retain residual protein function and complete constitutional loss of function is likely prenatally lethal (13).

Although the data presented here indicate that *PIGA* mutation is sufficient to disrupt GPI surface anchoring, the mechanisms by which this may contribute to duodenal adenoma or carcinoma development remains unclear. To further assess aberrant cellular processes in adenomas with *PIGA* mutations, we identified 88 genes that were overexpressed and 1,325 genes that were downregulated. We noted significant enrichment of DEGs associated with the plasma membrane, brush border, and the apical cellular compartment, including genes involved in absorption and digestion. The digestive brush-border enzyme *ALPI* is a marker of enterocytes and a GPI-AP downregulated in *PIGA*-mutant adenomas, indicating potential for enterocyte dedifferentiation to an immature crypt phenotype (19). This enzyme is also a component of the gut mucosal defense system, preventing bacterial translocation in the gut. Further to the transcriptional evidence provided here, work in PNH indicates that *PIGA* mutant cells may have a survival advantage. This may be intrinsic, due to a decreased response to apoptotic stimuli, or associated with an altered response to inflammatory stimuli, indicating an ability to evade or escape immune surveillance as a factor in clonal expansion (20).

Recent studies characterizing mutational processes in normal tissues are having an impact on our understanding of the roles of driver genes in tumorigenesis. With aging, many normal tissues accumulate mutations in established tumor driver genes that appear insufficient for tumorigenesis. Further genomic instability, additional driver events, changes in the microenvironment or the effects of inherited genotypes appear to be required for transformation. It may be that *PIGA* loss contributes to a more permissive environment for tumor progression through alteration of the cellular processes described above. Further exploration of the nature of clonal expansion of *PIGA* mutated cells in normal tissue and early and more advanced tumors, will be crucial to determining why this gene is recurrently mutated in duodenal adenomas and how dysregulation of GPI-anchors contributes to tumorigenesis.

## Supplementary Material

Supplementary Figure S1 Whole transcriptome sequencing quality control and read distribution for data sets from Cardiff and MD Anderson. A. Sequencing quality assessment, batch aggregates are shown. B. Read distribution across the genome per batch demonstrating that over 80% of reads mapped to known exons. CDS=Coding sequence; MDA=MD Anderson Cancer Center data; TES=Transcription end site; TSS=Transcription start site; UTR=Untranslated region.

Supplementary Figure S2 Somatic mutation rates in FAP and MAP-associated duodenal adenomas. A. Total, non-silent and silent mutation rate for all FAP and MAP adenomas. For FAP-associated duodenal adenomas, the average total mutation rate was 3.3 mutations per Mb (0.33-10.65 mutations/Mb), whilst for MAP adenomas, it was 4.9 mutations per Mb (0.94-15.1 mutations/Mb). The average non-silent mutational rate for FAP duodenal adenomas was 1.96 non-silent mutations/Mb (0.23-7.69 mutations/Mb), whilst for MAP adenomas it was 2.95 non-silent mutations/Mb (0.43-8.44 mutations/Mb). B. Differences in total, non-silent and silent mutational rates between the two diseases. There were no significant differences in total, non-silent and silent mutational rates between the two diseases. n.s. P>0.05; * P ≤0.05; ** P≤0.01; *** P ≤0.001.

Supplementary Figure S3 The gating tree was set as follows, shown for an organoid line without a PIGA somatic variant. A: FSC/SSC to isolate cells (represents the distribution of cells in the light scatter based on size and intracellular composition, respectively) to B: FSC-A/FSC-H (selects for single cells) to C: live gate (DRAQ7 negative, which represents the fraction of viable cells within the sample analysed) to D: SSC-A/FITC-A positive (selecting for FLAER positive cells). SSC-A/FITC-A gates of an unstained sample (E) and PIGA- sample (F) is included for comparison.

Supplementary Figure S4 RNASeq WTS pipeline validation by quantitative RT-PCR (qRT-PCR) and IHC A. Comparison of normalised RNA-Seq read counts (orange) to relative qRT-PCR expression levels (purple) for four top DEGs. Δ CT values are shown, with low values indicative of low mRNA abundances. High concordance between the two methods can be observed. qRT-PCR confirms increased levels of S100P and KRT7 and decreased levels of FCER2 and CA4 mRNA in duodenal adenomas in comparison to normal duodenal mucosa samples (purple bars). B. IHC results for S100 and CK7 expression in duodenal normal mucosa or duodenal adenoma tissue sections. Moderate and relatively strong positive staining for CK7 and S100, respectively, was also confirmed in duodenal adenomas compared to corresponding normal duodenal mucosa samples. Representative samples that had undergone RNA-Seq are shown. Scale bar: 50μm. COL=Colorectal samples; DUO=Duodenal samples

Supplementary Figure S5 Gene Ontology of DEGs between duodenal adenomas with and without PIGA somatic mutation. Enriched terms are plotted for Biological process(BP), A. Cellular Component (CC), B. and Molecular Function(MF), C. Terms are coloured by Log2-Padj value for term enrichment and sized by semantic similarity of GO term. DEGs for the most enriched terms; Apical plasma membrane (GO0016324), Salt transmembrane transporter activity (GO1901702), Organic anion transport (GO0015711), lipid transport (GO0006869) and the PPAR signalling pathway (KEGG03320) are plotted, D. Genes are coloured by Log2FC and sized by Log10 Padj. The central role of CD36 is highlighted.

Supplementary Table S1 Overview of all intestinal samples used in this study. *Sequenced previously (Thomas et al, 2017) and reanalysed here. Somatic mutations in APC, KRAS and PIGA as identified by WES and WTS analysis of CU and MDACC duodenal adenomas. Samples where matched WTS data was available are indicated with * and those where the variant was present in both WES and WTS data are denoted with **. 1All protein changes refer to the respective canonical isoforms. 2CADD is a meta-estimator for the deleteriousness of SNVs and indels in the human genome. The score given here ranks a variant relative to all possible substitutions of the genome on a logarithmic scale, i.e. a variant with a score >10 is predicted to be amongst the 10% most deleterious mutations possible, a variant with a score >20 indicates the 1% most deleterious. The developers suggest a cut-off of 15 to identify potentially pathogenic variants (Kircher et al, 2014). C=Colectomy (exact surgical procedure unspecified); Can=Cancer; CRC=Colorectal cancer; DUO=Duodenum; H=Homozygous; IPAA=Ileoanal pouch anastomosis; IRA=Ileo-rectal anastomosis; IS=Ileostomy; PC=Proctocolectomy; NA=Not available, CADD=Combined Annotation Dependent Depletion; DEL=Deletion; FS=Frameshift, INS=Insertion; SNV=Single nucleotide variant; VAF=Variant allele frequency. WES. Whole exome sequencing only. WES/WTS. Whole exome and whole transcriptome sequencing. WTS. Whole transcriptome sequencing only

Supplementary Table S2 Germline and somatic mutations in FAP patient derived duodenal organoids

Supplementary Table S3 Summary statistics for all FAP and MAP patient-derived intestinal samples analysed in this study. Data sets are from two centres, Cardiff University (CU) and MD Anderson Cancer Center (MDACC). Statistically significant P-values < 0.05 are in bold.

Supplementary Table S4 A VCF file containing putatively pathogenic variants found in 1142 intestinal cancer associated genes in 27 of the duodenal adenomas studied here (see supplementary table 1 for sample details). Full genomic datasets for these samples are available through EGA under accession: EGAD00001009332.

Supplementary Table S5 A VCF file containing putatively pathogenic variants found in 1142 intestinal cancer associated genes in 21 of the duodenal adenomas studied here (see supplementary table 1 for sample details). Full genomic datasets for these samples are available through EGA under accession: EGAD00001009332.
