# Supplementary figures and images for: PIGA Mutations and Glycosylphosphatidylinositol Anchor Dysregulation in Polyposis-Associated Duodenal Tumorigenesis
Source: Mol Cancer Res. 2024 Mar 28;22(6):515–23. doi: 10.1158/1541-7786.MCR-23-0810 (PMC11148540; doi:10.1158/1541-7786.MCR-23-0810)

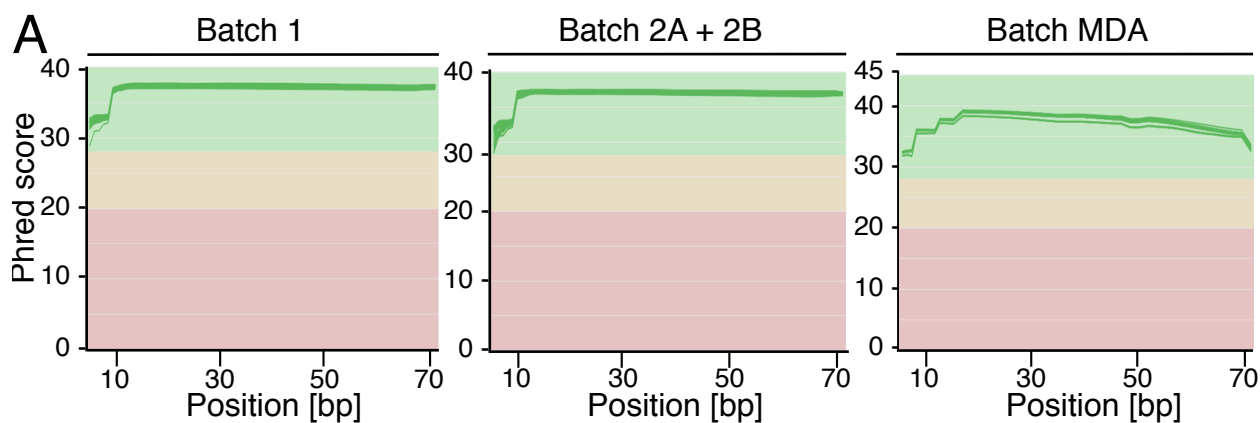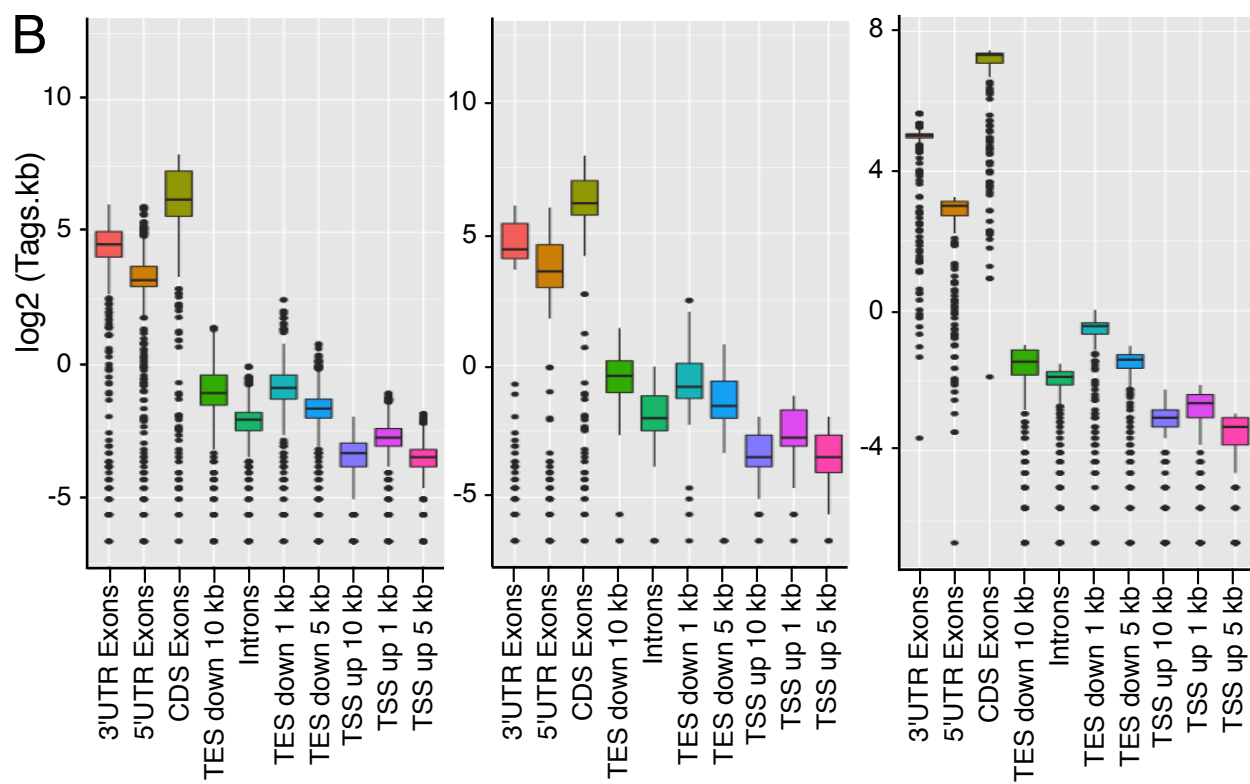

Supplement: Supplementary Figure S1 — Whole transcriptome sequencing quality control and read distribution for data sets from Cardiff and MD Anderson. A. Sequencing quality assessment, batch aggregates are shown. B. Read distribution across the genome per batch demonstrating that over 80% of reads mapped to known exons. CDS=Coding sequence; MDA=MD Anderson Cancer Center data; TES=Transcription end site; TSS=Transcription start site; UTR=Untranslated region. [file mcr-23-0810_supplementary_figure_s1_suppsf1.pdf]

# Mutation rates

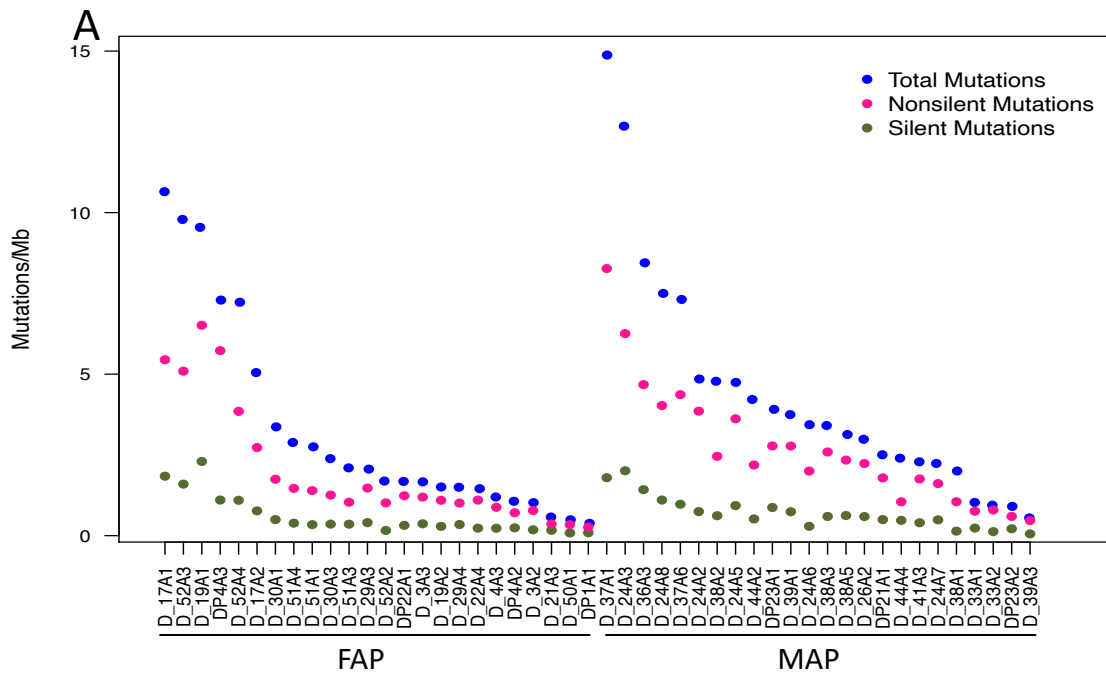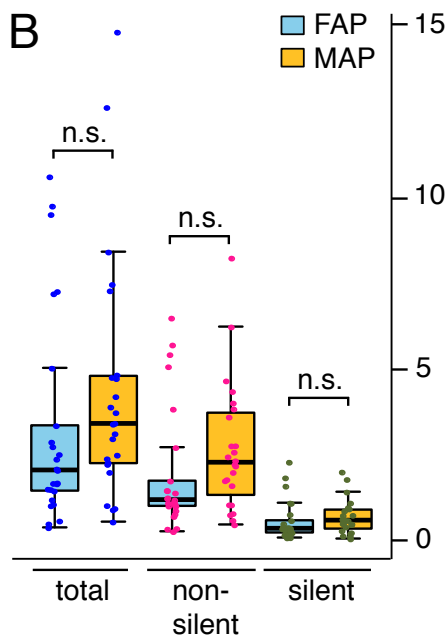

Supplement: Supplementary Figure S2 — Somatic mutation rates in FAP and MAP-associated duodenal adenomas. A. Total, non-silent and silent mutation rate for all FAP and MAP adenomas. For FAP-associated duodenal adenomas, the average total mutation rate was 3.3 mutations per Mb (0.33-10.65 mutations/Mb), whilst for MAP adenomas, it was 4.9 mutations per Mb (0.94-15.1 mutations/Mb). The average non-silent mutational rate for FAP duodenal adenomas was 1.96 non-silent mutations/Mb (0.23-7.69 mutations/Mb), whilst for MAP adenomas it was 2.95 non-silent mutations/Mb (0.43-8.44 mutations/Mb). B. Differences in total, non-silent and silent mutational rates between the two diseases. There were no significant differences in total, non-silent and silent mutational rates between the two diseases. n.s. P>0.05; * P ≤0.05; ** P≤0.01; *** P ≤0.001. [file mcr-23-0810_supplementary_figure_s2_suppsf2.pdf]

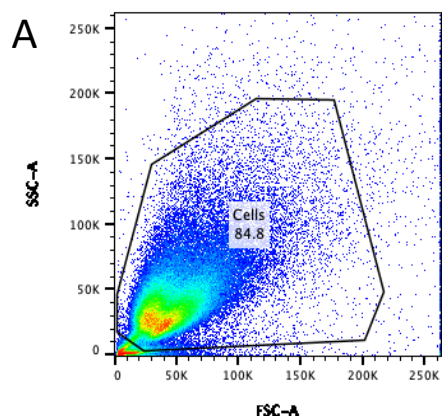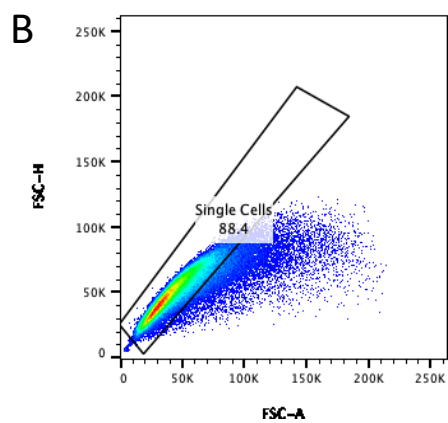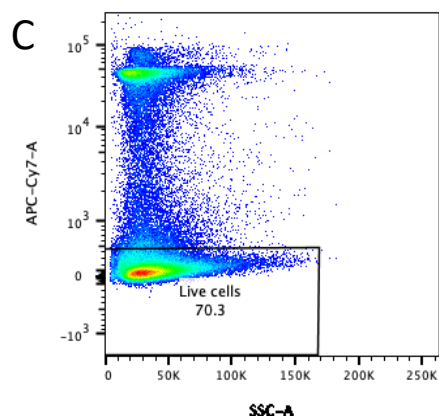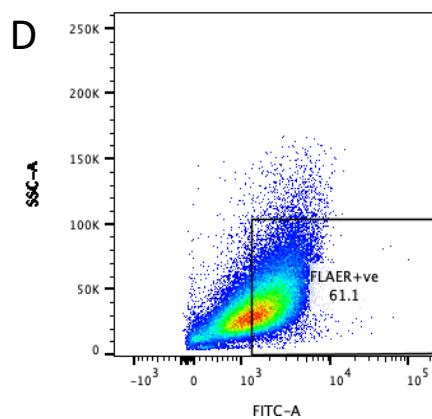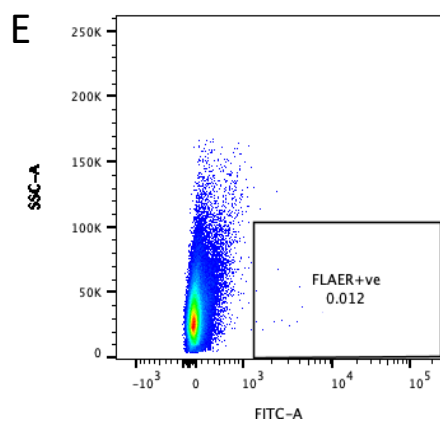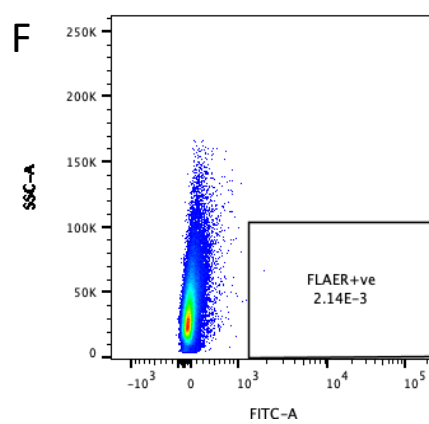

Supplement: Supplementary Figure S3 — The gating tree was set as follows, shown for an organoid line without a PIGA somatic variant. A: FSC/SSC to isolate cells (represents the distribution of cells in the light scatter based on size and intracellular composition, respectively) to B: FSC-A/FSC-H (selects for single cells) to C: live gate (DRAQ7 negative, which represents the fraction of viable cells within the sample analysed) to D: SSC-A/FITC-A positive (selecting for FLAER positive cells). SSC-A/FITC-A gates of an unstained sample (E) and PIGA- sample (F) is included for comparison. [file mcr-23-0810_supplementary_figure_s3_suppsf3.pdf]

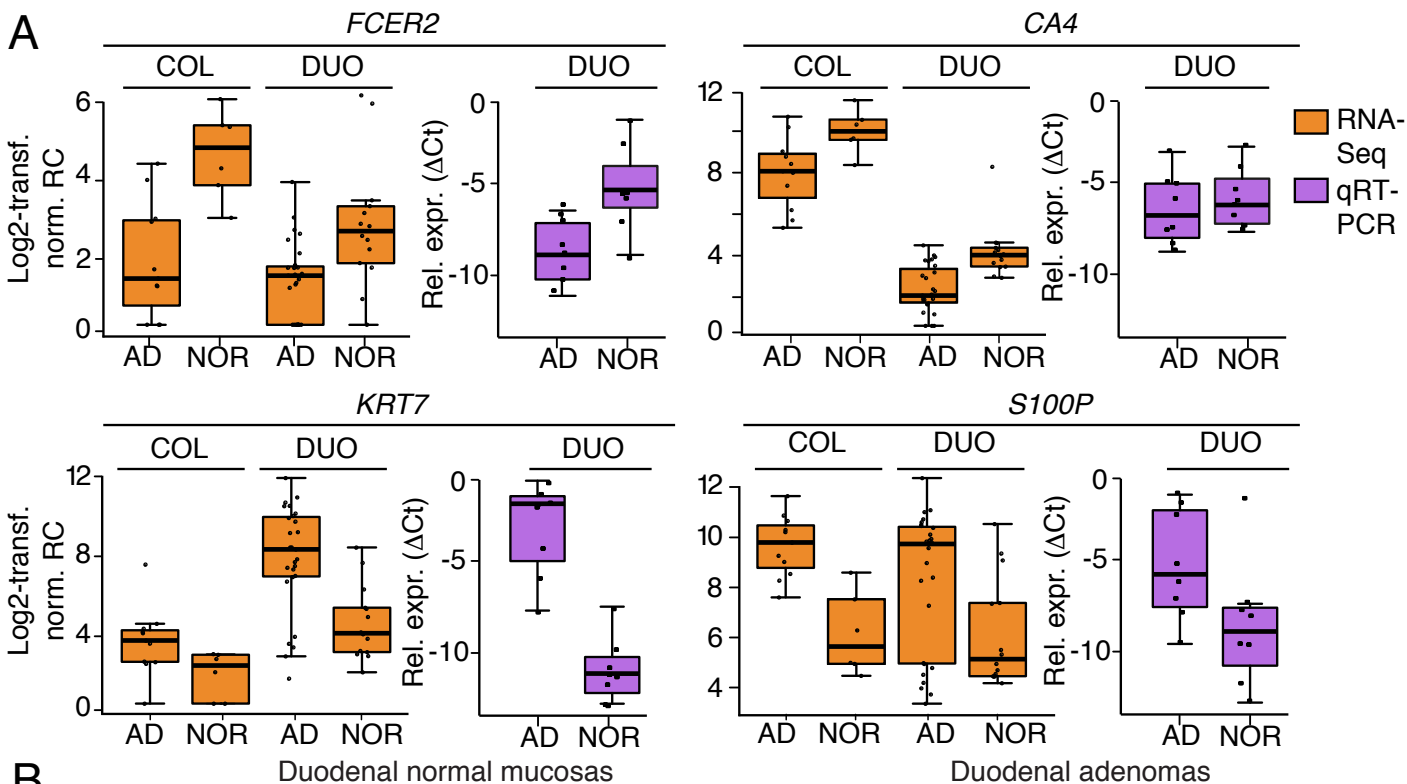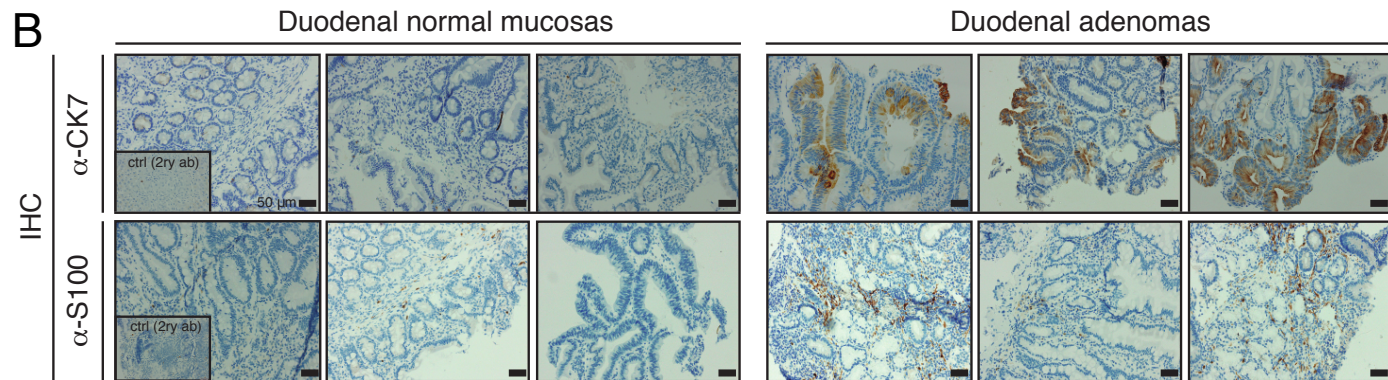

Supplement: Supplementary Figure S4 — RNASeq WTS pipeline validation by quantitative RT-PCR (qRT-PCR) and IHC A. Comparison of normalised RNA-Seq read counts (orange) to relative qRT-PCR expression levels (purple) for four top DEGs. Δ CT values are shown, with low values indicative of low mRNA abundances. High concordance between the two methods can be observed. qRT-PCR confirms increased levels of S100P and KRT7 and decreased levels of FCER2 and CA4 mRNA in duodenal adenomas in comparison to normal duodenal mucosa samples (purple bars). B. IHC results for S100 and CK7 expression in duodenal normal mucosa or duodenal adenoma tissue sections. Moderate and relatively strong positive staining for CK7 and S100, respectively, was also confirmed in duodenal adenomas compared to corresponding normal duodenal mucosa samples. Representative samples that had undergone RNA-Seq are shown. Scale bar: 50μm. COL=Colorectal samples; DUO=Duodenal samples [file mcr-23-0810_supplementary_figure_s4_suppsf4.pdf]

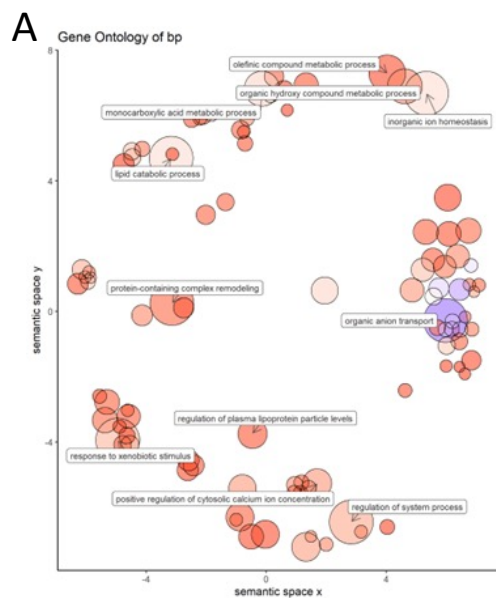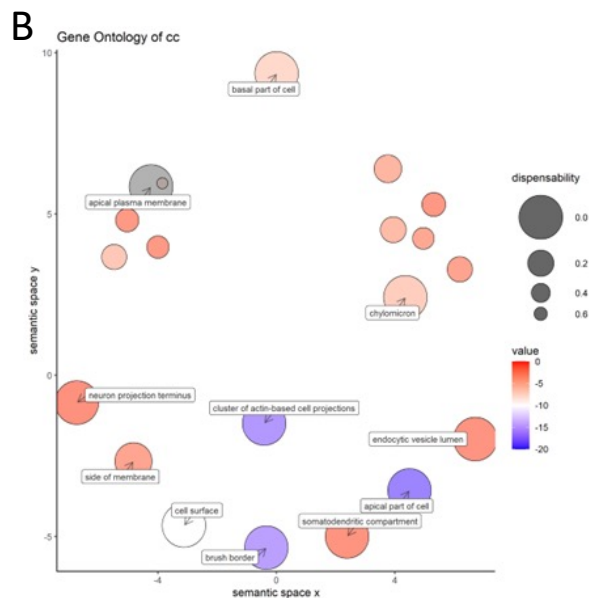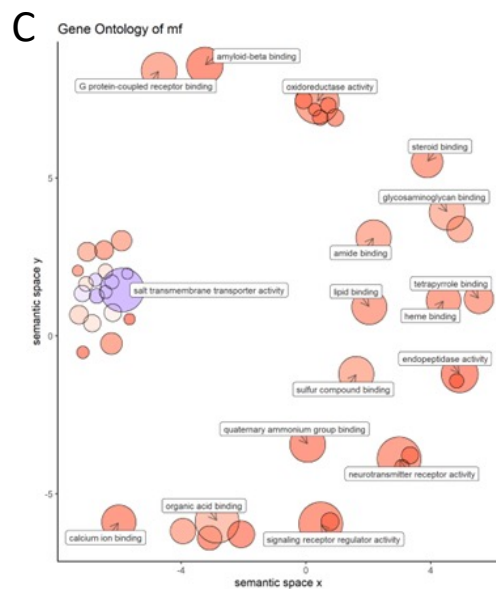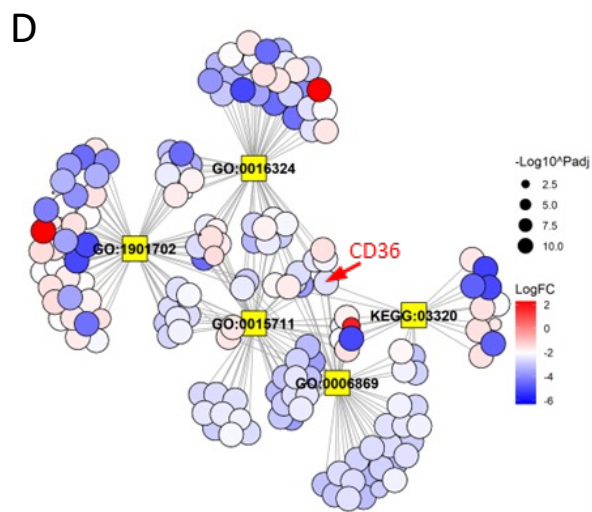

Supplement: Supplementary Figure S5 — Gene Ontology of DEGs between duodenal adenomas with and without PIGA somatic mutation. Enriched terms are plotted for Biological process(BP), A. Cellular Component (CC), B. and Molecular Function(MF), C. Terms are coloured by Log2-Padj value for term enrichment and sized by semantic similarity of GO term. DEGs for the most enriched terms; Apical plasma membrane (GO0016324), Salt transmembrane transporter activity (GO1901702), Organic anion transport (GO0015711), lipid transport (GO0006869) and the PPAR signalling pathway (KEGG03320) are plotted, D. Genes are coloured by Log2FC and sized by Log10 Padj. The central role of CD36 is highlighted. [file mcr-23-0810_supplementary_figure_s5_suppsf5.pdf]
